# The possibility to perform surgery in late posttraumatic gastrothorax only on history and clinical exam

**DOI:** 10.1186/1749-8090-8-S1-P43

**Published:** 2013-09-11

**Authors:** C Tunea, O Burlacu, G Cozma, V Voiculescu, I Miron, R Murza, A Nicodin

**Affiliations:** 1 Department of Thoracic Surgery, Emergency Municipal Hospital Timisoara, Romania; 2 University of Medicine, Timisoara, Romania

## Background

Due to possible severe complications, posttraumatic gastrothorax must be surgically corrected as soon as the diagnosis is reached. We tried to demonstrate that history and clinical exam can lead to the right diagnosis and consequently to the surgical repair.

## Method

We analyzed all cases with viscerothorax (8) admitted in 2002 - 2012. Four were gastrothorax. All the cases were clinically examined and at least one chest X-ray was taken with 4 of them having a CT.

## Results

The diagnosis based on history (4 minimal stab wounds, 3 politrauma, and 1 difficult nephrectomy) and clinical exam matched the diagnosis. In one case (difficult nephrectomy two years before) the radiologic interpretation led to a wrong diagnosis of tension pneumothorax and a chest tube was inserted with consecutive gastric perforation and leakage of gastric content. Promptly recognized, thoracotomy was performed like in all the other cases.

## Conclusions

It is difficult to diagnose a late gastrothorax when the traumatic event happened years ago and you base your diagnosis more on the radiological studies than on history and clinical exam. A careful history focusing on possible past traumas and a thorough clinical exam can give you the correct diagnosis.

